# The effects of lung and prostate cancer bone metastasis on serum osteoprotegerin levels: a meta-analysis

**DOI:** 10.1038/srep18324

**Published:** 2015-12-16

**Authors:** Leyuan Zang, Min Ma, Jianxin Hu, Hao Qiu, Bo Huang, Tongwei Chu

**Affiliations:** 1 Department of Orthopedics, Xinqiao Hospital, Third Military Medical University, Chongqing, 400037, China

## Abstract

Bone metastasis leads to skeletal-related events in final-stage cancer patients. The incidence of prostate and lung cancers increases yearly; these cancers readily invade the bone. Some recent studies have found that serum osteoprotegerin (OPG) levels may be altered in patients with bone metastasis, whereas other reports have produced inconsistent findings. Hence, we conducted a meta-analysis to examine the effects of lung and prostate cancer on serum OPG levels. A systematic literature search was conducted using PubMed, Medline, and CNKI to identify relevant studies. A total of 11 studies were included. The standardized mean difference (SMD) and 95% confidence interval (95% CI) of the bone metastasis (BM) group, the non-bone metastasis (BM-) group and healthy controls were calculated. In prostate cancer, serum OPG levels in the BM group were higher than in the BM- group and healthy controls. Additionally, no significant difference in serum OPG levels was found between the BM- group and healthy controls. In lung cancer, serum OPG levels in the BM and BM- groups were significantly increased compared with healthy controls. However, no significant difference in serum OPG levels was found between the BM and BM- groups. Studies with larger sample sizes are required to confirm these findings.

Bone metastases are often clinically manifested by patients with advanced malignant tumours and are most common in prostate and lung cancer patients^1^. In advanced lung cancer, metastatic deposits in bone are common and represent a source of pain and morbidity^2^. It is estimated that more than 35% of patients with advanced lung cancer manifest bone metastases, and a higher proportion was found in an autopsy series^3^. Similarly, as the second leading cause of cancer-related deaths in men, prostate cancer (PCa) has the ability to easily invade the bone, and more than 80% of PCa patients die from the development of bone metastasis^4^,^5^.

Bone metastasis incites bony destruction and skeletal-related events (SREs), such as ostealgia, pathological fracture and hypercalcaemia^6^. Among patients with bone metastasis, 45%–75% experience more pain due to secondary changes of bone metastasis^7^. Furthermore, bone metastasis results in a shorter survival time and worse prognosis^8^. Hence, the early detection of bone metastasis is critical for the clinical management and accurate staging of tumours^9^. However, only a fraction of bone metastasis cases are clinically diagnosed^10^.

Currently, the detection of bone metastasis mainly depends on pathology, imaging technologies and bone scans. However, most patients cannot tolerate undergoing a biopsy due to the invasiveness of the procedure. Each type of imaging technology has its own limitations: X-ray has hysteresis; CT has intense radiation; and MRI or PET/CT has a low incremental cost-effectiveness ratio. Although high-sensitivity bone scanning is widely used, the accuracy of its results is controversial due to low specificity. Hence, finding an effective and convenient detection method to diagnose bone metastasis is necessary.

It would be exciting to find a blood index that could accurately reflect bone metastasis. Recently, several new biomarkers of bone metabolism have been identified that can reflect bone turnover at an early stage. Some markers are already being widely applied in clinical practice^11^. However, the application of these biomarkers in the clinical diagnosis of bone metastases is not feasible^10^. Osteoprotegerin (OPG), also referred to as osteoclastogenesis inhibitory factor (OCIF), is a secreted glycoprotein that can suppress the function of osteoclasts (OCs); it was first observed in 1997^12^. As a soluble tumour necrosis factor receptor, OPG has become a research hotspot in many fields, including bone metabolism. In the same year, RANKL (receptor activator of nuclear factor-κB ligand), a ligand of OPG, was discovered^13^. RANK, the exclusive receptor for RANKL, is found in OCs. In bone metabolism, as a functional spindle^12^, OPG/RANK/RANKL regulates cell maturation and differentiation. Many studies have shown that lung and prostate cancer bone metastases can increase serum OPG levels. However, other studies have shown contradictory results. Hence, we conducted this meta-analysis of relevant studies to assess the effects of lung and prostate cancer bone metastasis on serum OPG levels.

## Methods

### Literature Search Strategy

An electronic literature search was executed in PubMed, Medline and CNKI (China National Knowledge Infrastructure, a widely used search engine in China) to identify relevant studies published up to June 2015. The following keywords or phrases were used: “OPG”, “osteoprotegerin”, “osteoclastogenesis inhibitory factor” and “OCIF” in combination with “(lung cancer) or (prostate cancer) bone metastasis”. We manually screened the reference lists of all eligible articles to obtain more studies.

### Inclusion Criteria

The inclusion criteria were as follows: 1. reports in English or Chinese; 2. cases of either primary malignancy or bone metastasis with definitive diagnoses and the collection of blood samples before the study subjects received any treatment measures; 3. the use of healthy or non-bone metastasis patients as controls; and 4. available data were supplied or obtainable through calculations.

### Exclusion Criteria

Studies were excluded if they met the following criteria: 1. the absence of a case-control design; 2. review articles, case reports or conference articles; 3. animal or *in vitro* studies; 4. research involving bone primary tumours; and 5. research involving special populations (e.g., infants or pregnant women).

### Study Selection

Initially, we reviewed the titles and abstracts to identify potential studies that fulfilled the inclusion criteria. In cases of uncertainty regarding the relevance of a report, a subsequent full-text assessment was conducted. Because the data used for this study were retrieved from published literature, we did not need to obtain approval from an ethics committee.

### Data Extraction

Two authors independently extracted the following information: author, publication year, nationality, mean age, sample size, and serum OPG levels (mean ± SD).

### Validity Assessment

Based on the primary criteria for non-randomized and observational studies of the Newcastle-Ottawa Quality Assessment scale (NOS) for meta-analyses^14^, two authors completed the quality assessment. Disagreements were resolved by discussion.

### Statistical Analysis

The analysis was performed with Stata 12.0 and Review Manager 5.2 software. Standard mean differences (SMDs) and the corresponding 95% confidence intervals (CIs) were used to measure serum OPG levels. Homogeneity testing was performed using the I^2^ statistic. In the absence of heterogeneity (I^2^ ≤ 50%), a fixed-effects model was used to combine the SMDs. In the opposite case, a random-effects model was used. Additionally, we performed a sensitivity analysis to assess the effect of a single study on the overall estimate by rejecting each study one at a time. Furthermore, publication bias was detected using Begg’s and Egger’s tests. p-values less than 0.05 were considered statistically significant.

## Results

### Study screening process

**Figure 1:**
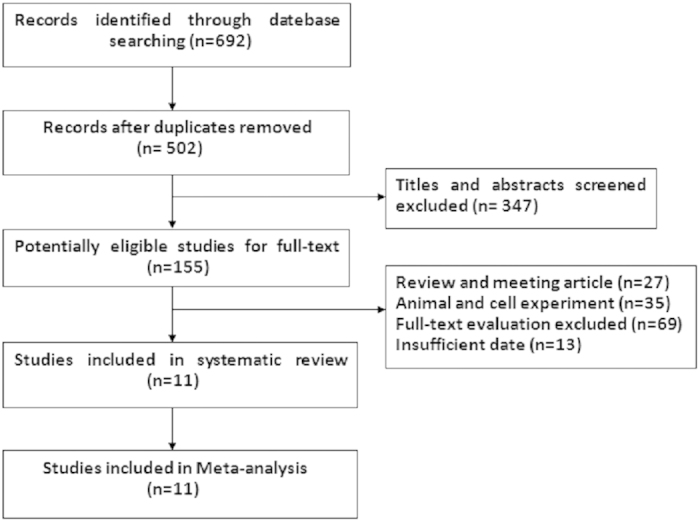
Flow chart of the studies identified, included and excluded.

We initially identified 692 potential studies from the above databases. Most were excluded because they were not case-control studies or had incomplete data. Finally, 11 studies (PCa, 6; lung cancer, 5) were included^15^,^16^,^17^,^18^,^19^,^20^,^21^,^22^,^23^,^24^,^25^ (Fig. 1). These 11 eligible studies involved 448 cases of BM (PCa, 213; lung cancer, 235), 409 BM- (PCa, 215; lung cancer, 194) and 349 healthy controls. The general data from the 11 studies are summarised in Table 1 and Table 2.

### The effect of prostate cancer bone metastasis on serum OPG levels

**Figure 2:**
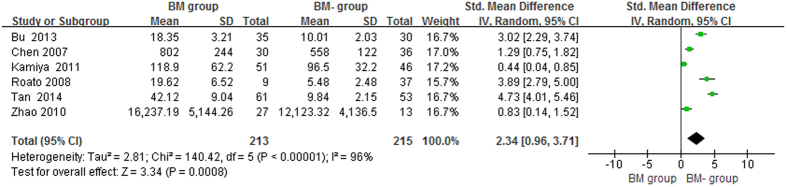
Forest plot for the comparison of serum OPG levels between BM group and BM- group in prostate cancer.

**Figure 3:**
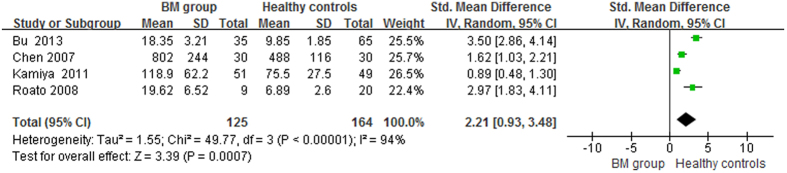
Forest plot for the comparison of serum OPG levels between BM group and healthy controls in prostate cancer.

**Figure 4:**
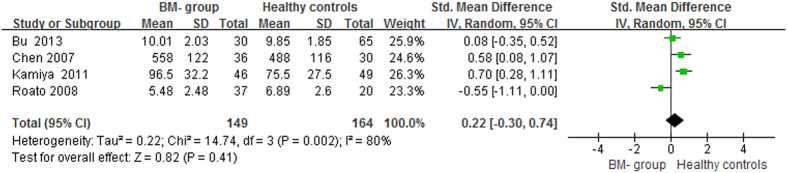
Forest plot for the comparison of serum OPG levels between BM- group and healthy controls in prostate cancer.

The pooled data suggested that the serum OPG levels in the BM group were significantly higher than in the BM- group and healthy controls, with pooled SMDs (95% CIs) of 2.34 (0.96, 3.71) and 2.21 (0.93, 3.48), p < 0.05, respectively (Figs 2 and 3). However, no significant difference in serum OPG levels was found between the BM- group and healthy controls (SMD = 0.22, 95% CI = −0.30 to 0.74, p = 0.41, >0.05; Fig. 4).

**Figure 5:**
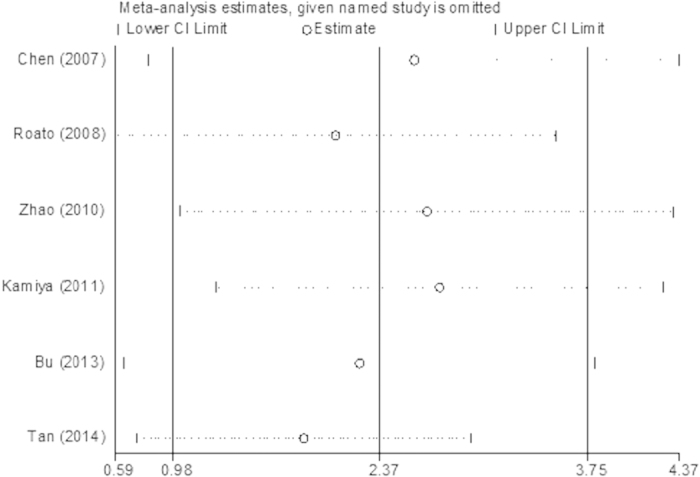
The plot of sensitivity analysis for the comparison of serum OPG levels between BM group and BM- group in prostate cancer.

We conducted a sensitivity analysis regarding the comparison of serum OPG levels between the BM and BM- groups by sequentially eliminating one study from the relevant data; Stata 12.0 software was used to pool the SMD for the remaining studies. The results consistently suggested that no single study significantly altered the combined results (Fig. 5). Egger’s regression test indicated little evidence of publication bias (p > 0.05).

### The effect of lung cancer bone metastasis on serum OPG levels

**Figure 6:**
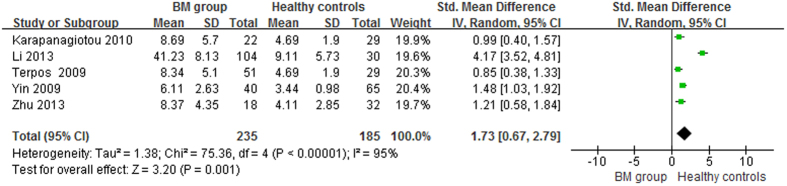
Forest plot for the comparison of serum OPG levels between BM group and healthy controls in lung cancer.

**Figure 7:**
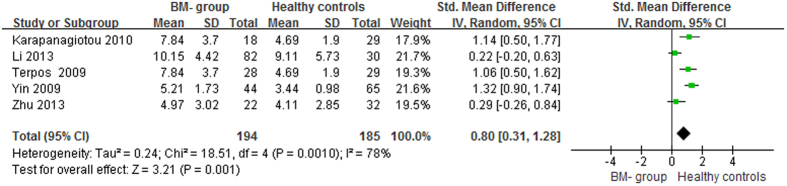
Forest plot for the comparison of serum OPG levels between BM- group and healthy controls in lung cancer.

**Figure 8:**
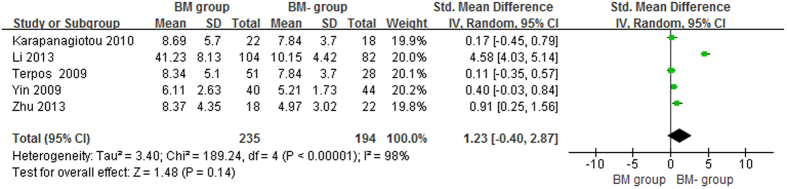
Forest plot for the comparison of serum OPG levels between BM group and BM- group in lung cancer.

The lung cancer results were different from the prostate cancer results: compared with healthy controls, the serum OPG levels in the BM and BM- groups were significantly higher (p < 0.05), with pooled SMDs (95% CIs) of 1.73 (0.67, 2.79) and 0.80 (0.31, 1.28), respectively (Figs 6 and 7). However, no significant difference was found in serum OPG levels between the BM and BM- groups (SMD = 1.23; 95% CI = −0.40 to 2.87; p = 0.14, >0.05; Fig. 8).

**Figure 9:**
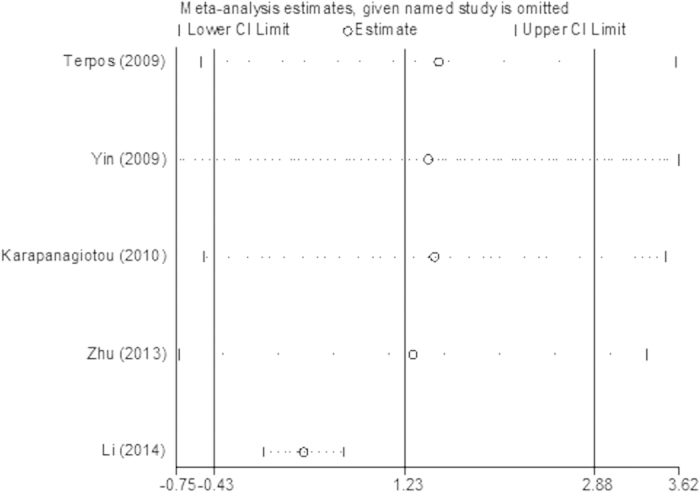
The plot of sensitivity analysis for the comparison of serum OPG levels between BM group and BM- group in lung cancer.

**Figure 10:**
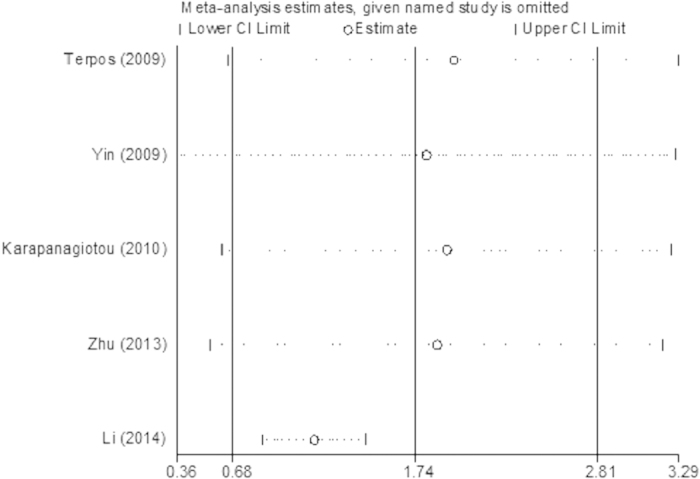
The plot of sensitivity analysis for the comparison of serum OPG levels between BM group and healthy controls in lung cancer.

**Figure 11:**
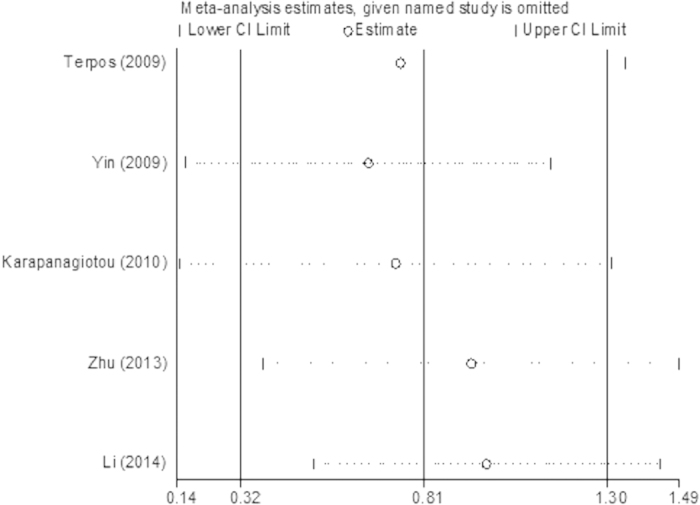
The plot of sensitivity analysis for the comparison of serum OPG levels between BM- group and healthy controls in lung cancer.

We used Stata 12.0 software to perform the sensitivity analysis. No single study changed the combined results significantly, which indicated that the results were statistically stable and reliable (Figs 9,10 and 11). Egger’s regression test indicated little evidence of publication bias (p > 0.05).

## Discussion

Metastasis represents a major cause of mortality in cancer patients, and bone invasion is often described. Generally, lung and prostate cancers exhibit high levels of bone tropism^2^,^26^. To explain the cancer metastasis phenomenon, Stephen Paget proposed the “seed and soil” hypothesis in 1889, which suggests that the interplay between the properties of cancer cells and the particular organ microenvironment determines the selective growth advantage of cells^27^. Recently, most emerging evidence emphasises the crucial role of feedback interactions between tumour cells and the bone microenvironment, which lead to the establishment of a vicious cycle that acts by upregulating the physiological mechanisms that normally favour bone resorption.

Although the osteoclasia caused by bone metastases is divided into osteolytic and osteoblastic types, mixed lesions are often described. Much evidence has shown that both resorption and formation are activated in most bone metastases; therefore, both osteolytic and osteoblastic characteristics can be observed^2^. Osteolytic and osteoblastic metastases are only the two extremes, however. The characteristics of a lesion are mainly based on the balance between resorption and formation in the bone environment. Activation and dysregulation exist in most osseous lesions, resulting in an unbalanced bone remodelling process and reflected in the complex phenotypic outcome^28^,^29^,^30^.

OCs and osteoblasts play an important role in maintaining the balance of bone remodelling. Osteolytic metastases are thought to be caused by factors secreted by tumour cells, which activate OCs^30^. Osteoblastic metastases are believed to be caused by osteoblasts producing factors that stimulate osteoblast proliferation, differentiation and, therefore, bone formation.

Many researchers have reported that tumour cells mainly express RANKL when they adhere to the bone microenvironment^31^,^32^. In general, RANKL can bind to RANK to trigger signal transduction and thus promote the differentiation and maturity of OC precursors. OPG acts in this case as a “decoy” receptor of RANKL and could be considered a “protector” of bone^33^. As a paracrine regulator of OC formation, OPG was found to have an essential physiological role: OPG is produced by osteoblasts and binds to RANKL, and its constitutive production is necessary to limit the OC formation resulting from RANKL stimulation^34^.

The RANK/RANKL/OPG system has recently been recognised as the ultimate mediator of osteoclastogenesis^30^,^35^, and the dysregulation of the OPG–RANKL system is thought to be crucial to the bone disease connected with cancers such as prostate cancer^36^. Some studies have reported that the ratio of RANKL/OPG was increased in neoplastic disease patients with severe osteolysis, which has also been observed in multiple myeloma patients^37^,^38^. Recently, some studies showed that a therapy that disrupts the vicious cycle in the bone microenvironment by binding to RANKL and inhibiting its function could achieve satisfactory results. Denosumab is a fully humanised monoclonal antibody against RANKL for the prevention of SREs in patients with tumours that metastasize to bone^39^. Unlike denosumab, zoledronic acid (ZA) can prevent the prenylation of the small GTPase proteins that are essential for OC function and survival^40^,^41^. In 2013, Sun found that denosumab was superior to ZA in preventing complications in patients with bone metastases^42^. One year later, Henry reported that denosumab was more effective than ZA at either delaying SREs in solid bone tumours or preventing pain progression^43^. These results suggest that the RANK/RANKL/OPG system plays a key role in the process of bone metastasis from the perspective of treatment; thus, serum OPG levels could be altered in bone metastasis patients.

In our study, the pooled data on PCa bone metastasis revealed that, compared with the BM- group and healthy controls, the BM group had higher serum OPG levels. However, no difference was found between the BM- group and healthy controls. The results suggest that bone metastasis increases serum OPG levels in prostate cancer patients. The specific mechanisms underlying the increase in serum OPG levels might be due to a variety of factors. The OC is a unique cell capable of dissolving bone tissue, which plays an important role in bone remodelling. It has been shown that OPG can be secreted to inhibit osteoclastogenesis and OC survival, thus preventing the establishment of tumour lesions in bone^44^,^45^,^46^. *In vitro* studies have shown that some tumour cells did not express RANKL mRNA when cultured alone. However, the co-culture of these cancer cells with osteoblastic cells or bone marrow stromal cells could induce RANKL expression. This interaction between stromal cells and tumour cells is critical for metastasis^29^,^47^. OPG can be released to upset the interaction between OCs and stromal cells^48^, which inhibits RANKL expression by tumour cells. Despite the potential of OPG in inhibiting OC activation, it also binds to TNF-related apoptosis-inducing ligand (TRAIL), making tumour cells resistant to apoptosis. It was shown that prostate cancer and bone marrow stromal cells could express OPG to enhance tumour cell survival by inhibiting TRAIL^36^,^49^,^50^. OPG can prevent the association between TRAIL and its death-inducing receptor, thereby increasing the survival of tumour cells that have metastasised to the bone microenvironment^29^,^51^,^52^; this may be one reason that OPG levels increase when tumours metastasise to bone.

However, we found that bone metastasis embodied a different phenomenon in lung cancer. Compared with healthy controls, the BM and BM- groups had higher serum OPG levels. No difference was found between the BM group and the BM- group. Serum OPG levels are increased in lung cancer regardless of whether it is complicated by bone metastases. The literature on the role of OPG and its involvement in metastatic bone disease is somewhat contradictory. This may suggest that OPG is not associated with skeletal metastasis at all, but rather with the tumour load per se^17^. In addition, preclinical data suggest that OPG plays a role in promoting angiogenesis^53^, which may be a less important reason for OPG expression in lung cancer bone metastasis.

The meta-analysis revealed that serum OPG levels could reflect prostate cancer bone metastasis. This has a certain clinical utility in that abnormally elevated serum OPG levels in prostate cancer patients may be associated with bone metastases. Serum OPG measurements can be used to supplement various existing diagnostic methods to increase the precision and convenience of bone metastasis diagnosis. However, we found that OPG was not associated with lung cancer bone metastasis, but rather with the presence of the tumour. This finding perhaps hints that not all bone metastases can be detected by measuring serum OPG. Recently, a study of bone metastasis treatment showed that clinical efficacy was consistent with the relative reduction observed in bone turnover markers^43^. This may suggest that OPG could be used as an indicator to evaluate the clinical efficacy of treatment.

We should note some limitations of our study: 1. the included studies were based on a case-control design, in which selection bias was inevitable; 2. the sample size of the included studies was relatively small, and several studies were excluded due to insufficient data; 3. we could not avoid the possibility that other unmeasured or inadequately measured factors confounded the results; and 4. our inclusion criteria might have introduced selection bias, although little statistical evidence of publication bias was observed.

## Conclusion

The present meta-analysis suggests that serum OPG levels can reflect prostate cancer bone metastasis to some extent, which indicates that serum OPG measurements can be used to supplement existing diagnostic methods for bone metastasis. However, serum OPG levels may more accurately reflect the existence of lung cancer itself. Further studies on this topic with larger sample sizes are needed.

## Additional Information

**How to cite this article**: Zang, L. *et al.* The effects of lung and prostate cancer bone metastasis on serum osteoprotegerin levels: a meta-analysis. *Sci. Rep.* **5**, 18324; doi: 10.1038/srep18324 (2015).

## Figures and Tables

**Table 1:** The characteristics of included 6 prostate cancer bone metastasis studies.

Author	Year	Country	Mean age*	Sample Size*
Chen *et al.*	2007	China	68/70/48	30/36/30
Roato *et al.*	2008	Italy	67/64/60	9/37/20
Zhao *et al.*	2010	China	N/N/N	27/13/N
Kamiya *et al.*	2011	Japan	69.2/66.2/62.9	51/101/49
Bu	2013	China	65.2/63.8/43.5	35/30/65
Tan	2014	China	45.5/45.8/N	61/53/N

Note: *: Bone metastasis group/non-bone metastasis group/healthy controls.

**Table 2:** The characteristics of included 5 lung cancer bone metastasis studies.

Author	Year	Country	Mean age*	Sample Size*
Terpos *et al.*	2009	Greece	66.6/69.6/65.3	51/28/29
Yin *et al.*	2009	China	N/N/53	40/44/65
Karapanagiotou.et al.	2010	Greece	59.6/64.3/54.8	22/18/29
Zhu *et al.*	2013	China	N/N/66	18/22/32
Li *et al.*	2013	China	N/N/N	104/82/30

Note: *: Bone metastasis group/non-bone metastasis group/healthy controls.

## References

[b1] Riccio A. I., Wodajo F. M. & Malawer M. Metastatic carcinoma of the long bones. Am. Fam. Physician 76, 1489–1494 (2007). 18052014

[b2] Mundy G. R. Metastasis to bone: causes, consequences and therapeutic opportunities. Nat. Rev. Cancer 2, 584–593 (2002). 12154351 10.1038/nrc867

[b3] Yin J. J., Pollock C. B. & Kelly K. Mechanisms of cancer metastasis to the bone. Cell Res. 15, 57–62 (2005). 15686629 10.1038/sj.cr.7290266

[b4] Keller E. T. *et al.* Prostate carcinoma skeletal metastases: cross-talk between tumor and bone. Cancer Metastasis Rev. 20, 333–349 (2001). 12085970 10.1023/a:1015599831232

[b5] Bubendorf L. *et al.* Metastatic patterns of prostate cancer: an autopsy study of 1,589 patients. Hum. Pathol. 31, 578–583 (2000). 10836297 10.1053/hp.2000.6698

[b6] Coleman R. E. Skeletal complications of malignancy. Cancer 80, 1558–1594 (1997). 10.1002/(sici)1097-0142(19971015)80:8+<1588::aid-cncr9>3.3.co;2-z 9362426

[b7] Furukawa M., Ohta T. & Xiong Y. Activation of UBC5 ubiquitin-conjugating enzyme by the RING finger of ROC1 and assembly of active ubiquitin li-gases by all cullins. J. Biol. Chem. 277, 15758–15765 (2002). 11861641 10.1074/jbc.M108565200

[b8] Destombe C. *et al.* Investigation for bone metastasis from an unknown primary. Joint Bone Spine 74, 85–89 (2007). 17218141 10.1016/j.jbspin.2006.05.009

[b9] Voorzanger-Rousselot N. *et al.* Association of 12 serum biochemical markers of angiogenesis, tumour invasion and bone turnover with bone metastases from breast cancer: a crossectional and longitudinal evaluation. Br. J. Cancer 95, 506–514 (2006). 16880790 10.1038/sj.bjc.6603285 PMC2360666

[b10] Zhao H. *et al.* Value of C-telopeptide-cross- linked Type I collagen, osteocalcin, bone-specific alkaline phosphatase and procollagen Type I N-terminal propeptide in the diagnosis and prognosis of bone metastasis in patients with malignant tumors. Med. Sci. Monit. 17, CR626–CR633 (2011). 22037741 10.12659/MSM.882047 PMC3539492

[b11] Fontana A. & Delmas P. D. Markers of bone turnover in bone metastases. Cancer 88, 2952–2960 (2000). 10898339 10.1002/1097-0142(20000615)88:12+<2952::aid-cncr11>3.0.co;2-m

[b12] Penno H. *et al.* Osteoprotegerin secretion from prostate cancer isstimulated by cytokines, *in vitro*. Biochem. Biophys. Res. Commun. 293, 451–455 (2002). 12054622 10.1016/S0006-291X(02)00242-5

[b13] Simonet W. S. *et al.* Osteoprotegerin: a novel secreted protein involved in the regulation of bone density. Cell 89, 309–319 (1997). 9108485 10.1016/s0092-8674(00)80209-3

[b14] Stang A. Critical evaluation of the Newcastle-Ottawa scale for the assessment of the quality of non-randomized studies in meta-analyses. Eur. J. Epidemiol. 25, 603–605 (2010). 20652370 10.1007/s10654-010-9491-z

[b15] Chen H. X. *et al.* Serum osteoprotegerin as a novel marker of bone metastasis in prostate cancer. Chin. J. Surg. 45, 412–414 (2007). 17537330

[b16] Roato I. *et al.* Osteoclasts are active in bone forming metastases of prostate cancer patients. Plos One 3, e3627 (2008). 18978943 10.1371/journal.pone.0003627 PMC2574033

[b17] Terpos E. *et al.* The clinical significance of serum markers of bone turnover in NSCLC patients: surveillance, management and prognostic implications. Anticancer Res. 29, 1651–1658 (2009). 19443381

[b18] Yin J. L. & Chen X. Y. The clinical value of serum osteoprotegerin level in the diagnosis of lung cancer. Exp. Lab. Med. 27, 7–9 (2009).

[b19] Zhao X. Z. *et al.* Clinical value of measuring serum osteoprotegerin in patients with prostate cancer. Clinical Medicine of China 26, 1242–1243 (2010).

[b20] Karapanagiotou E. M. *et al.* Serum bone turnover markers may be involved in the metastatic potential of lung cancer patients. Med. Oncol. 27, 332–338 (2010). 19373566 10.1007/s12032-009-9214-z

[b21] Kamiya N. *et al.* Significance of serum osteoprotegerin and receptor activator of nuclear factor κB ligand in Japanese prostate cancer patients with bone metastasis. Int. J. Clin. Oncol. 16, 366–372 (2011). 21327451 10.1007/s10147-011-0193-7

[b22] Zhu B., Shao K. J., Yuan H. & Xu Y. F. The clinical assessment on lesions shown ^99m^Tc-MDP whole body bone scan and determination of serum levels of OPG, BSP and TRACP-5b in lung cancerpatients with bone metastasis. J. Radioimmunology. 26, 545–547 (2013).

[b23] Bu J. S. Changes and clinical significance of serum OPG, BMP-7 and PSA in patients with prostatic cancer. Chinese Journal of Health Laboratory Technology 23, 168–170 (2013).

[b24] Li L., Tan B. Y., Liu Z. W., Chen M. & Zhang Y. The diagnostic value of serum osteoclast differentiation factor and inhibitory factor in bone metastasis of lung cancer. Int. J. Lab. Med. 34, 1930–1934 (2013).

[b25] Tan Y. C. Significance of ODF and OCIF in diagnosis of prostate cancer bone metastases. China Journal of Modern Medicine 24, 37–40 (2014).

[b26] Schaller B. *et al.* Prostate-specific antigen in the cerebrospinal fluid leads to diagnosis of solitary cauda equina metastasis: a unique case report and review of the literature. Br. J. Cancer 77, 2386–2389 (1998). 9649164 10.1038/bjc.1998.397 PMC2150374

[b27] Paget S. The distribution of secondary growths in cancer of the breast. Cancer Metastasis Rev. 8, 98–101 (1989). 2673568

[b28] Gregory R. & Mundy M. D. Mechanisms of bone metastasis. Cancer 80, 1546- 1556 (1997). 9362421 10.1002/(sici)1097-0142(19971015)80:8+<1546::aid-cncr4>3.3.co;2-r

[b29] Neville-Webbe H. L. *et al.* Osteoprotegerin (OPG) produced by bone marrow stromal cells protects breast cancer cells from TRAIL-induced apoptosis. Breast Cancer Res. Treat. 86, 269–279 (2004). 15567943 10.1023/b:brea.0000036900.48763.b3

[b30] Hofbauer L. C. & Schoppet M. Clinical implications of the osteoprotegerin/ RANKL/RANK system for bone and vascular diseases. JAMA 292, 490–495 (2004). 15280347 10.1001/jama.292.4.490

[b31] Nagai M., Kyakumoto S. & Sato N. Cancer cells responsible for humoral hypercalcaemia express mRNA encoding a secreted form of ODF/TRANCE that induces osteoclast formation. Biochem. Biophys. Res. Commun. 269, 532–536 (2000). 10708588 10.1006/bbrc.2000.2314

[b32] Huang L., Cheng Y. Y., Chow L. T., Kumta S. M. & Zheng M. H. Tumor cells produce receptor activator of NF-kB ligand (RANKL) in skeletal metastases. J. Clin. Pathol. 55, 877–878 (2002). 12401833 10.1136/jcp.55.11.877 PMC1769799

[b33] Jung K. *et al.* Osteoprotegerin in serum as a novel marker of bone metastatic spread in prostate cancer. Clin. Chem. 47, 2061–2063 (2001). 11673385

[b34] Udawela M., Hay D. L. & Sexton P. M. The receptor activity modifying protein family of G protein coupled receptor accessory proteins. Semin. Cell Dev. Biol. 15, 299–308 (2004). 15125893 10.1016/j.semcdb.2003.12.019

[b35] Theoleyre S. *et al.* The molecular triad OPG/RANK/RANKL: involvement in the orchestration of pathophysiological bone remodelling. Cytokine Growth Factor Rev. 15, 457–475 (2004). 15561602 10.1016/j.cytogfr.2004.06.004

[b36] Holen I. & Shipman C. M. Role of osteoprotegerin (OPG) in cancer. Clin. Sci. 110, 279–291 (2006). 16464170 10.1042/CS20050175

[b37] Grimaud E. *et al.* Receptor activator of nuclear factor kappaB ligand (RANKL)/osteoprotegerin (OPG) ratio is increased in severe osteolysis. Am. J. Pathol. 163, 2021–2031 (2003). 14578201 10.1016/s0002-9440(10)63560-2 PMC1892410

[b38] Terpos E. *et al.* Soluble receptor activator of nuclear factor kappa-B ligand- osteoprotegerin ratio predicts survival in multiple myeloma: proposal for a novel prognostic index. Blood 102, 1064–1069 (2003). 12689925 10.1182/blood-2003-02-0380

[b39] Rosen L. S. *et al.* Zoledronic acid versus placebo in the treatment of skeletal metastases in patients with lung cancer and other solid tumors: a phase III, double-blind, randomized trial--the Zoledronic Acid Lung Cancer and Other Solid Tumors Study Group. J. Clin. Oncol. 21, 3150–3157 (2003). 12915606 10.1200/JCO.2003.04.105

[b40] Russell R. G., Watts N. B., Ebetino F. H. & Rogers M. J. Mechanisms of action of bisphosphonates: similarities and differences and their potential influence on clinical efficacy. Osteoporos. Int. 19, 733–759 (2008). 18214569 10.1007/s00198-007-0540-8

[b41] Rogers M. J., Crockett J. C., Coxon F. P. & Mönkkönen J. Biochemical and molecular mechanisms of action of bisphosphonates. Bone 49, 34–41 (2010). 21111853 10.1016/j.bone.2010.11.008

[b42] Sun L. & Yu S. Efficacy and safety of denosumab versus zoledronic acid in patients with bone metastases: a systematic review and meta-analysis. Am. J. Clin. Oncol. 36, 399–403 (2013). 22772430 10.1097/COC.0b013e31824be20e

[b43] Henry D. *et al.* Delaying skeletal-related events in a randomized phase 3 study of denosumab versus zoledronic acid in patients with advanced cancer: an analysis of data from patients with solid tumors. Support Care Cancer 22, 679–87 (2014). 24162260 10.1007/s00520-013-2022-1

[b44] Zhang J. *et al.* Osteoprotegerin inhibits prostate cancer induced osteoclastogenesis and prevents tumor growth in the bone. J. Clin. Invest. 107, 1235–1244 (2001). 11375413 10.1172/JCI11685 PMC209296

[b45] Miller R. E. *et al.* RANK ligand inhibition plus docetaxel improves survival and reduces tumor burden in a murine model of prostate cancer bone metastasis. Mol. Cancer Ther. 7, 2160–2169 (2008). 18606716 10.1158/1535-7163.MCT-08-0046

[b46] Hakeda Y. *et al.* Osteoclastogenesis inhibitory factor (OCIF) directly inhibits bone-resorbing activity of isolated mature osteoclasts. Biochem. Biophys. Res. Commun. 251, 796–801 (1998). 9790989 10.1006/bbrc.1998.9523

[b47] Reddi A. H., Roodman D., Freeman C. & Mohla S. Mechanisms of tumor metastasis to the bone: challenges and opportunities. J. Bone Miner. Res. 18, 190–194 (2003). 12568395 10.1359/jbmr.2003.18.2.190

[b48] Akatsu T. *et al.* Osteoclastogenesis inhibitory factor suppresses osteoclast survival by interfering in the interaction of stromal cells with osteoclast. Biochem. Biophys. Res. Commun. 250, 229–234 (1998). 9753612 10.1006/bbrc.1998.9294

[b49] Holen I., Crouche P. I., Hamdy F. C. & Eaton C. L. Osteoprotegerin (OPG) is a survival factor for human prostate cancer cells. Cancer Res. 62, 1619–1623 (2002). 11912131

[b50] Nyambo R. *et al.* Human bone marrow stromal cells protect prostate cancer cells from TRAIL-induced apoptosis. J. Bone Miner. Res. 191, 712–1721 (2004). 10.1359/JBMR.040703 15355567

[b51] Park H. R. *et al.* Expression of osteoprotegerin and RANK ligand in breast cancer bone metastasis. J. Korean Med. Sci. 18, 541–546 (2003). 12923331 10.3346/jkms.2003.18.4.541 PMC3055084

[b52] Holen I. *et al.* Osteoprotegerin (OPG) expression by breast cancer cells *in vitro* and breast tumours *in vivo*-a role in tumour cell survival? Breast Cancer Res. Treat. 92, 207–215 (2005). 16155791 10.1007/s10549-005-2419-8

[b53] Cross S. S. *et al.* Osteoprotegerin (OPG)-a potential new role inthe regulation of endothelial cell phenotype and tumour angiogenesis? Int. J. Cancer 118, 1901–1908 (2006). 16287088 10.1002/ijc.21606

